# Aerobic Fitness in Children and Young Adults with Primary Ciliary Dyskinesia

**DOI:** 10.1371/journal.pone.0071409

**Published:** 2013-08-19

**Authors:** Astrid Madsen, Kent Green, Frederik Buchvald, Birgitte Hanel, Kim Gjerum Nielsen

**Affiliations:** Danish PCD Centre and Pediatrics Pulmonary Service, Department of Pediatrics and Adolescent Medicine, Copenhagen University Hospital, Rigshospitalet, Denmark; University of Giessen Lung Center, Germany

## Abstract

**Background:**

Although aerobic fitness is regarded as an overall prognostic measure of morbidity and mortality, its evaluation in the chronic progressive sinopulmonary disease primary ciliary dyskinesia (PCD) has been infrequently and inconsistently reported. Here we assessed peak oxygen uptake (VO_2peak_) in a large well-characterized cohort of PCD patients, and explored whether VO_2peak_ was associated with parameters of pulmonary function, self-reported physical limitations, and physical activity level.

**Methods:**

VO_2peak_, spirometry, diffusing capacity, whole-body plethysmography, and nitrogen multiple breath inert gas washout (N_2_ MBW) were assessed in a cross-sectional, single-occasion study of clinically stable children and young adults with PCD. We used a questionnaire including self-reported physical limitations in everyday life or in vigorous activities, and estimation of weekly hours of strenuous physical activity. VO_2peak_ in PCD patients was compared with that in matched, healthy control subjects and a national reference.

**Results:**

Forty-four PCD patients aged 6–29 years exhibited reduced VO_2peak_ compared to healthy controls (*P*<0.001) and the national reference. VO_2peak_ was abnormal (z-score <–1.96) in 34% of PCD patients. Spirometric values, RV/TLC, and indices of N_2_ MBW were significantly abnormal, but VO_2peak_ only correlated with FEV_1_ and DL_CO_/V_A_. VO_2peak_ correlated with complaints of moderate or significant limitations in vigorous activities (*P* = 0.0001), exhibited by 39% of PCD patients.

**Conclusion:**

One-third of PCD patients exhibited substantially lower aerobic fitness than healthy subjects. Aerobic fitness correlated with FEV_1_, DL_CO_/V_A_ and self-reported complaints of limitations in vigorous physical activity. These findings are most likely explained by PCD pulmonary disease and its impact on pulmonary function and physical ability. Considering fitness as an important outcome and including regular strenuous physical activity in PCD treatment would probably altogether increase pulmonary clearance, lung function, aerobic fitness, and quality of life, and prevent lifestyle-related diseases.

## Introduction

The peak oxygen uptake (VO_2peak_) test is widely used to objectively determine a person's aerobic fitness or cardiopulmonary functional capacity, and as a prognostic measure of morbidity and mortality in disease and in health [1], [2]. VO_2peak_ reflects the ability to perform sustained exercise and thus can be used in patients with chronic pulmonary disease, to provide information that can not be obtained from standard pulmonary function tests [1]. In cystic fibrosis (CF), VO_2peak_ is significantly correlated with survival and quality of life [3], [4], leading to recommendations of annual assessment [4].

Primary ciliary dyskinesia (PCD) is a rare autosomal recessive disorder that affects approximately 1 in 20.000 individuals and is characterized by immotile or dyskinetic respiratory cilia [5]. PCD and CF share several features, including impaired clearance of the lower airways that leads to recurrent and chronic pulmonary infections, and inevitably progresses through declining lung function and bronchiectasis to chronic respiratory failure [6]. PCD causes significant morbidity and impaired quality of life, including limitations of physical activity [7], [8]. In preschool-age children PCD can already impose a serious threat to lung function [9], although there exists a high degree of variation in the course of lung function after diagnosis [10]. Furthermore, the vast majority of children and adolescents receiving regular centralized care still exhibit substantial peripheral airway dysfunction, as reflected in indices of SF_6_ multiple breath inert gas washout (MBW) [11].

Pulmonary function is traditionally assumed to be an important independent predictor of disease morbidity, disease control, and severity, and it may have an impact on aerobic fitness. However, it remains unclear whether VO_2peak_ differs between PCD patients with normal spirometry vs. abnormal spirometry, and healthy controls or if it is associated with other measures of pulmonary function. To date, only two small studies have partly investigated these questions using spirometric measures, and have reported contradictory results. Valerio et al. [12] found impaired VO_2peak_ in PCD patients with FEV_1_ below 85% of the predicted value, compared to matched healthy controls: they have also found that male gender, age, and time spent in vigorous activity were independent predictors of aerobic fitness. In contrast, Wells et al. [13] showed no difference in VO_2peak_ between adolescents with PCD and healthy controls.

The primary aim of the present study was to assess VO_2peak_ in a large cohort of children and young adults with PCD and to compare these values with those of healthy subjects. Secondly, we aimed to evaluate the association between VO_2peak_ and a panel of relevant pulmonary function measures, as well as self-reported physical limitations and weekly physical activity.

Part of this study was previously presented in abstract form at the American Thoracic Society (ATS) International Conference 2012, San Francisco (Abstract no. 30080 presented May 21).

## Materials and Methods

### Ethics Statement

This study was approved by the research ethics committee of The Capital Region of Denmark (J.no. H-1-2010-042 and amendment 2013-37202). Verbal and written consent was obtained from all patients and healthy controls, as well as from parents or guardians on the behalf of the study participants who were <18 years of age.

### Study subjects

Children and young adults, (6–29 years of age) with documented PCD from the National Danish PCD center were eligible for the study. Healthy age-, gender- and BMI-matched non-atopic subjects with normal spirometry were included as controls. Subjects were excluded if they were unable to cooperate with the exercises or pulmonary function testing, e.g., due to mental or physical disability or known cardiovascular disease. PCD diagnosis was based on characteristic clinical symptoms [6], abnormal low nasal nitric oxide (nNO) measurement [14], repeated high-speed video microscopic recordings of abnormal ciliary beat pattern and/or frequency, and transmission electron microscopy analysis of ciliary ultrastructure according to previously published guidelines [5].

### Study design

We performed a cross-sectional, single-occasion, and single-center case-control study. In the following order, the PCD patients performed N_2_ MBW, spirometry, whole-body plethysmography, single-breath diffusing capacity for carbon monoxide (DL_CO_) and VO_2peak_ test. The matched healthy controls performed only spirometry and VO_2peak._ All tests for all participants were scheduled to be performed on a single occasion. Visits were postponed in the event of concurrent self-reported pulmonary exacerbation, or FEV_1_ decrease of more than 10 percentage points compared to the last visit at which the patient was considered clinically stable.

### Methods

#### Peak oxygen uptake (VO_2peak_)

The VO_2peak_ test was performed as described by Godfrey [15] with step increments of 10, 15, or 20 watts (W) per minute based on standing height. To achieve optimal test duration, the initial workload was determined according to the patient's heart rate while warming up [1]. A valid peak test was defined by continuous objective signs of exhaustion during verbal encouragement from the test leader, *combined* with at least one of the following criteria: respiratory exchange ratio (RER)>1.00 at test termination [16], or maximal heart rate (HR_max_) >85% of age-based predicted maximum (208-0.7× age) [17]. O_2_ and CO_2_ concentrations were analyzed using a mass spectrometer (Amis 2000, Innovision, Odense, Denmark). VO_2peak_ was calculated as ml/kg/min using the procedure described in the in supporting information, Text S1. We also calculated the ventilatory reserve (VR) reflecting ventilatory capacity, and the ventilatory equivalent of CO_2_ (V_E_/VCO_2_) reflecting efficiency of ventilation. VR <15% or V_E_/VCO_2_ >40 were considered abnormal and to be positive signs of ventilatory limitation during the test [18]. Reference values of VO_2peak_ were derived from comparable assessments in 937 healthy Danish children and young adults (426 males and 511 females) (Physical activity – Prevention and treatment) [19], [20] and this reference material was evaluated and compared with the group of matched healthy controls.

#### Pulmonary function tests

Spirometry, whole-body plethysmography, and DL_CO_ measurement were performed using Jaeger Master Screen Pro (CareFusion, Hochberg, Germany) according to ATS and ERS recommendations [21], [22], [23]. The “all-ages” reference equations were used for spirometry [24]. For children, the reference equations of Koopman et al. [25] were used for DL_CO_ and Zapletal [26] for whole-body plethysmography, except plethysmographic specific airway resistance (sRaw) for which we used the reference equations of Kirkby et al. [27]. For adults (>18 years), we used the reference equations of Cotes et al. [28] and Quanjer et al. [29] for DL_CO_ and whole-body plethysmography, respectively.

N_2_ MBW was performed using Exhalyzer D (Eco Medics AG, Duernten, Switzerland), which was completed prior to any tests requiring forced expiratory maneuvers [30] and at least one hour before the VO_2peak_ test. We calculated indices of N_2_ MBW, i.e. the Lung Clearance Index (LCI) and the normalized phase III slope indices S_cond_ and S_acin_ [30]. Pre-reviewed normative data was used as reference material [31].

#### Questionnaire

There is currently no validated PCD-specific instrument to assess quality of life or physical activity. Therefore, we selected and combined validated questions from the St George's Respiratory Questionnaire (SGRQ) [32], Cystic Fibrosis Questionnaire (CFQ-R) [33], Sino –Nasal Outcome Test-22 (SNOT-22) [34], and the Medical Outcomes Study Short Form-36 (SF-36) [35] and finally extracted simple questions about physical activity and limitations useful, particularly, for this study. All, including healthy control subjects, answered questions on the following subjects: physical limitations in activities of every-day-life due to symptoms; subjective judgment of the difficulty performing vigorous activities; and weekly hours spent on physical activities, such as running, cycling, and sports. The specific questions and the scoring system are shown in supplemental material, Text S2.

### Analysis

Where appropriate, data regarding all assessed lung function parameters and aerobic fitness are reported as median (range) or mean (SD) of absolute values, values in percent predicted or z-scores. The main outcomes were z-scores for VO_2peak_, FEV_1_, DL_CO_/V_A_, LCI and scores of self-reported physical limitations and level of weekly physical activity. Abnormal lung function and VO_2peak_ was defined as a z score <−1.96, whereas abnormal LCI was defined as z score >1.96. SAS Enterprise guide 4.3 (SAS institute, Cary, North Carolina, USA) and MedCalc© Version 12.3.0. (MedCalc Software, Mariakerke, Belgium) were used for statistical analyses.

The Mann-Whitney test was used to test the significance of the differences in various parameters between PCDs and healthy controls, and the Chi-square test for the comparison of proportions between these groups. Since VO_2peak_ is directly dependent on body weight and, hence, significantly correlated to BMI, we chose to correct for the latter. The associations between the z-scores for VO_2peak_, FEV_1_, and DL_CO_/V_A_ adjusted for BMI were analyzed with multiple regression analysis, using stepwise forward selection with an entry significance level of 0.05.

We used partial correlations to assess whether our sample size would provide adequate statistical power to show that one covariate (e.g. FEV_1_ z-scores) was a significant predictor of VO_2peak_, z-scores when controlling for the other covariates (e.g. DL_CO_/V_A_ and BMI, z-scores) using a Type III F test.

To detect a difference of 8.0 ml/kg/min between subgroups of PCD patients, the minimal required sample size per group was 15 assuming a SD of 6.6 ml/kg/min [12], with alpha  = 0.05 and beta  = 0.1. A two-tailed *P* value <0.05 was considered significant.

## Results

### Patient characteristics

**Figure 1:**
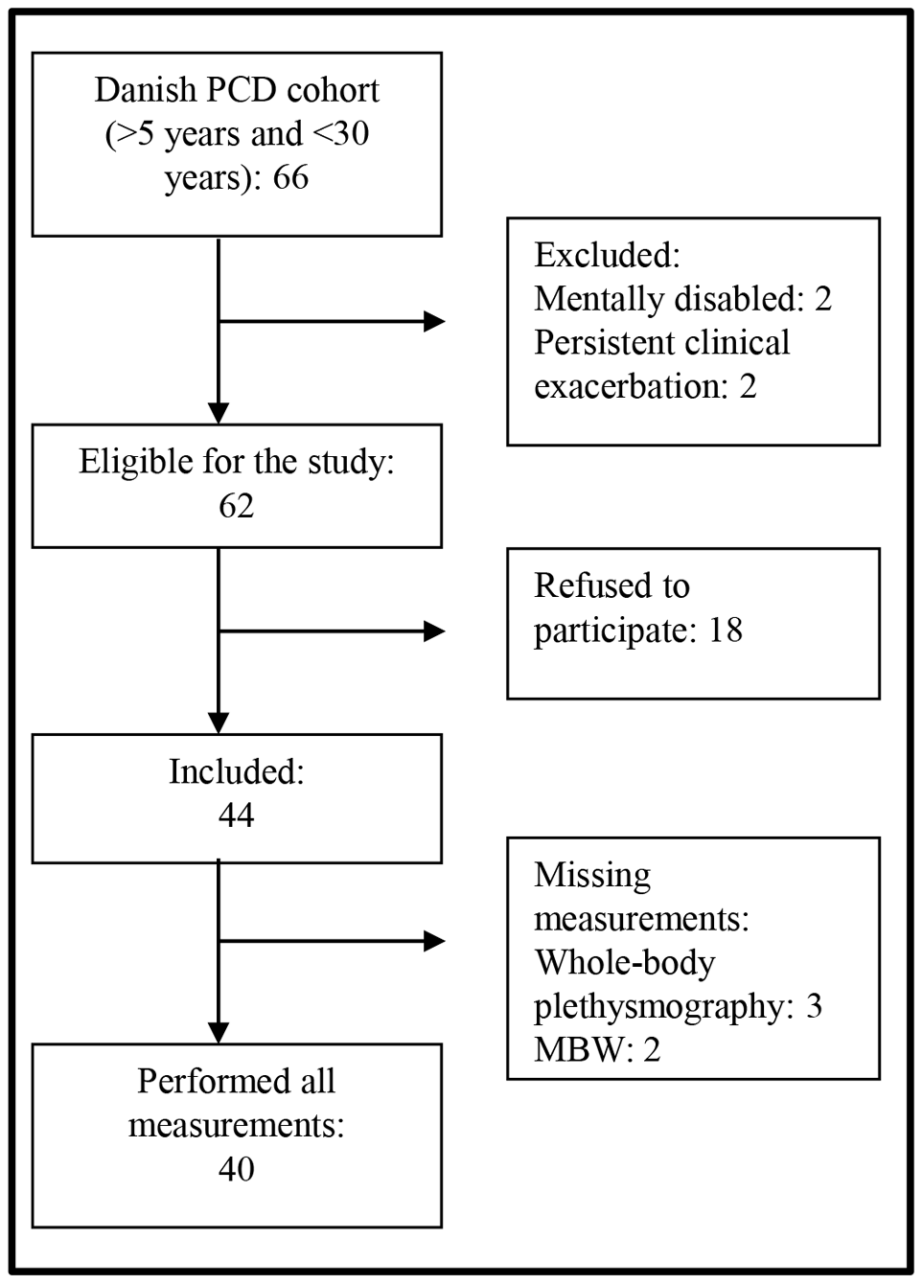
Flow chart showing the inclusion and investigation process.

From a total of 108 PCD patients in the Danish PCD cohort, 66 were eligible for the study and 67% (44/66) agreed to participate and were included during the study period. Figure 1 shows the inclusion flowchart. Table 1 shows the baseline and diagnostic characteristics. The gender ratio did not differ significantly from that in the background population (*P* = 0.08). The 18 patients who refused to participate did not differ in baseline demographics or spirometric data (data not shown). All but one patient performed the VO_2peak_ test and the pulmonary function tests on the same day, except one patient who performed the VO_2peak_ test one week later than the other tests due to technical problems. One patient did not complete the questionnaire.

**Table 1:** Demographic and diagnostic characteristics of patients with primary ciliary dyskinesia.

	Median (range)
**Demographics**	
N	44
Gender (M/F)	17/27
Age in years, median (range)	14.8 (6.5 to 29.7)
BMI in kg/m^2^, median (range)	19.9 (13.7 to 39.0)
BMI z-score, median (range)	−0.1 (−1.8 to 3.1)
Situs inversus	14
**Diagnostic tests**	
*Typical clinical symptoms*	44
*nNO performed*	44
Low level of nNO$	42
*Cilia beat pattern performed*	42
Immotility	16
Asynchrony	26
Not performed	2
*Electron microscopy performed*	39
Outer Dynein Arm (ODA) defect	11
Inner Dynein Arm (IDA) defect	1
ODA + IDA defect	7
Radial spoke defect	6
Transposition defect	2
Peripheral microtubule defect	6
Central microtubule defect	2
Normal with Hydin mutation [57]	4
Missing or not performed	5
**Gram-negative infections**	
Chronic PSA^†^	4
Intermittent PSA*	6
Chronic XA^§^	1

$ Median (range) nNO  = 26 (4 to 190);^ †^Chronic PSA  =  chronic infection with *Pseudomonas Aeruginosa*, defined as more than 50% of positive airway cultures the previous year; *Intermittent PSA  =  intermittent infection with *P. Aeruginosa*, defined as least one positive culture in the last year; ^§^Chronic AX  =  chronic infection with *Achromobacter xylosoxidans*, defined as more than 50% of positive airway cultures the previous year.

### Characteristics of healthy controls

Median age and BMI z-score of the 33 healthy controls were 14.4 years (range, 6.2 to 28.8 years) and, 0.0 (range, −2.2 to 2.8), respectively. The gender ratio (M/F) was 17/16. These values were not significantly different from those in the PCD patient group. Twenty-eight healthy control subjects completed the questionnaire.

### Pulmonary function parameters

In the PCD patients, abnormal z-scores were found in 27% (FEV_1_), 14% (FVC), 48% (FEF_25–75_) and 7% (TLC). Diffusion parameters were abnormal in only 7% (DL_CO_) and 2% (DL_CO_/V_A_), while median N_2_ MBW indices were highly increased and abnormal, specifically LCI in 93% (39/42). Table 2 shows the details of pulmonary function in PCDs patients.

**Table 2:** Baseline pulmonary function in patients with primary ciliary dyskinesia.

Functional parameters	% of predicted		z-score	
	Median	Range	Median	Range
**Spirometry (N = 44)**				
FEV_1_	84.9	52.9 to 112.8	−1.2	−4.1 to 1.3
FVC	97.0	72.0 to 115	−0.2	−2.6 to 1.5
FEF_25–75_	58.2	21.8 to 120.0	−1.9	−4.5 to 1.1
FEV_1_/FVC,	88.0	67.3 to 105.3	−1.4	−3.0 to 0.9
**Diffusion capacity (N = 44)**				
DL_CO_	94.1	73.2 to 135.4	−0.4	−2.3 to 2.3
DL_CO_/V_A_	95.8	74.3 to 128.0	−0.3	−2.1 to 2.5
**Multiple breath washout, N_2_ (N = 42)**				
LCI_N2_	157.1	98.0 to 255.3	12.2	−0.4 to 28.9
S_cond_	344.4	92.3 to 636.2	6.4	−0.2 to 14.1
S_acin_	220.1	61.2 to 812.4	2.7	−0.9 to 17.3
FRC_N2_, L	122.7	78.4 to 190.6	2.4	1.2 to 5.9
**Whole-body plethysmography (N = 41)**				
sRaw (age <18 years), (N = 23)	109.8	74.2 to 495.2	0.4	0.9 to 6.2
FRC	107.4	78.2 to 155.2	0.4	−1.4 to 5.0
RV	140.2	63.7 to 211.2	1.8	−1.7 to 5.2
TLC	105.0	77.3 to 131.5	0.5	−2.6 to 4.8
VC	92.5	64.8 to 162.6	−0.7	−3.5 to 5.6
RV/TLC z-score	134.7	79.9 to 189.6	1.5	−1.3 to 5.6

FEV_1_: Forced Expiratory Volume in 1 s. FVC: Forced Vital Capacity. FEF_25–75_: Forced Mid Expiratory Flow. DL_CO_: Single Breath diffusing capacity of the lung for carbon monoxide. DL_CO_/V_A_: Diffusing Capacity corrected for Alveolar Volume. MBW, N_2_: Nitrogen Multiple Breath Washout LCI: Lung Clearance Index. S_cond_: Conductive Airways ventilatory heterogeneity. S_acin_: Acinar airways ventilatory heterogeneity. FRC: Functional Residual Capacity. sRaw: specific airway resistance. RV: Residual Volume. TLC: Total Lung Capacity. VC: Vital Capacity.

As per inclusion criteria, all healthy controls had normal spirometric values with the following median z-scores: FEV_1_, 0.7 (range, −0.8 to 4.4); FVC, 0.9 (range, −1.1 to 3.6), and FEF_25–75_, −0.1 (range, −2.8 to 3.4).

### VO_2peak_

**Figure 2:**
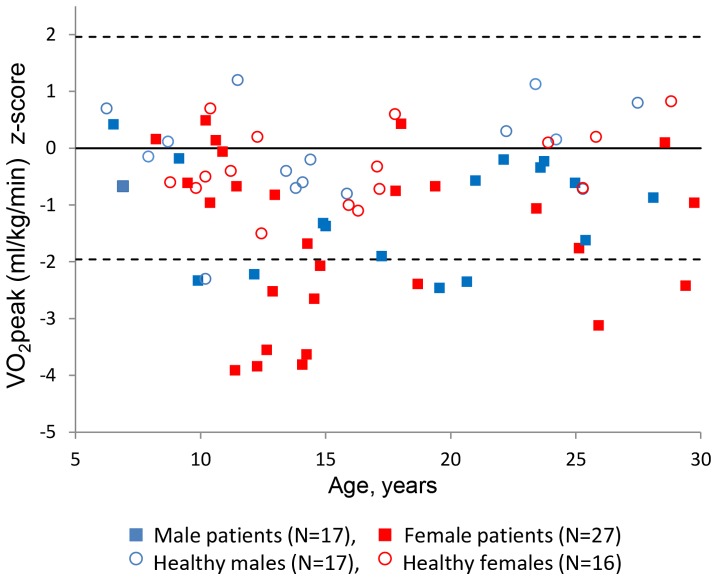
VO_2peak_ z-scores in 44 Danish PCD patients and 33 healthy control subjects according to age distribution. Solid line indicates a z-score of 0, and dashed lines indicate z-scores ±1.96.

All patients and healthy controls completed the exercise test and fulfilled the overall criteria for maximal performance. One patient did not reach HR >85% of predicted, and RER was below 1.0 in two other patients. The national reference material embraced all healthy control subjects except one (Figure 2). The results are tabulated in Table 3.

**Table 3:** Exercise test results in 44 PCD patients and 33 healthy subjects.

	PCD patients (N = 44)		Healthy subjects (N = 33)	
	Median	Range	Median	Range
VO_2peak,_ ml/kg/min	37.9***	19.4 to 51.0	44.4	30.6 to 57.6
VO_2peak_, ml/kg/min % predicted	83.7**	48.9 to 105.7	95.7	66.2 to 120.1
VO_2peak_, ml/kg/min, z-scores	−1.00***	−3.9 to 0.5	−0.3	−2.3 to 1.2
V_E_, L/min (BTPS)	65.9	38.6 to 163.1	93.8	40.7 to 185.5
FR, min^−1^	47	25 to 61	47.9	29.2 to 66.5
VT, L	1.5	0.8 to 3.0	1.9	0.9 to 3.8
RER	1.14***	0.95 to 1.26	1.23	1.05 to 1.36
HR_max_, bpm	192*	160 to 212	196	180 to 210
Min. S_p,O2_, %	97	90 to 100	97	94 to 100
Test duration, min.	6.1***	4.1 to 10.0	7.50	5.30 to 11.10
O2 pulse, % predicted	84.7**	50.4 to 108.2	95.9	62.9 to 126.0
W_max_, watt	135	70 to 330	210	60 to 420
W_max_/kg	3.1***	1.5 to 4.5	3.9	2.8 to 6.1
VR; %	27	−11.3 to 54.5	28.5	1.4 to 50.0
V_E_/VCO_2_, %	36.0**	26.6 to 48.8	33.2	26.5 to 43.4
AT, % VO_2peak_	57.5	29.5 to 74.4	61.3	39.0 to 75.4

VO_2peak_: Peak Oxygen uptake. V_E_: peak Minute Ventilation. RF: Respiratory Frequency. VT: Tidal Volume. RER: Respiratory Exchange Ratio. HR_max_: maximal Heart Rate. Min S_p_O_2_: oxygen saturation. W_max_: Maximal work load. W_max_/Kg: watts per kilogram.VR: Ventilatory Reserve (1-(Minute Ventilation/Maximal Voluntary Ventilation)*100). V_E_/VCO_2_: Ventilatory equivalent for CO_2_. AT, %VO_2_: Anaerobic Threshold (AT) % of pred.

* Patients with PCD vs. Healthy subjects (*P*<0.05), **Patients with PCD vs. Healthy subjects (*P*<0.01), ***Patients with PCD vs. Healthy subjects (*P*<0.001).

VO_2peak_ was significantly reduced in PCD patients compared to in healthy controls (Table 3) and when compared to the national reference material (*P*<0.001) exhibiting a median VO_2peak_ z-score of −1.00 (range, −3.90 to 0.50) (Figure 2), 34% of PCD patients (15/44) had abnormal VO_2peak_. Maximal heart rate, test duration, oxygen pulse, and maximum workload corrected for body weight (W_max_/kg) were each also significantly lower in PCD patients (Table 3). VO_2peak_ did not differ between male and female PCD patients (*P* = 0.18).

Among the PCD patients VR was decreased (<15%) in 27% (12/44) and V_E_/VCO_2_ was increased (>40) in 16% (7/44). V_E_/VCO_2_ was significantly increased in the total group of PCD patients, indicating ventilatory limitation during the test. However, of the patients with an abnormal VO_2peak_ (z-score <−1.96), only 20% (3/15) showed reduced VR, and increased V_E_/VCO_2_ was only seen in 27% (4/15). Desaturation (Sp,O_2_ <90%) or bronchial obstruction (decline of >12% in FEV_1_) did not occur in any patients during or after the exercise test.

### VO_2peak_ and association with lung function parameters

**Figure 3:**
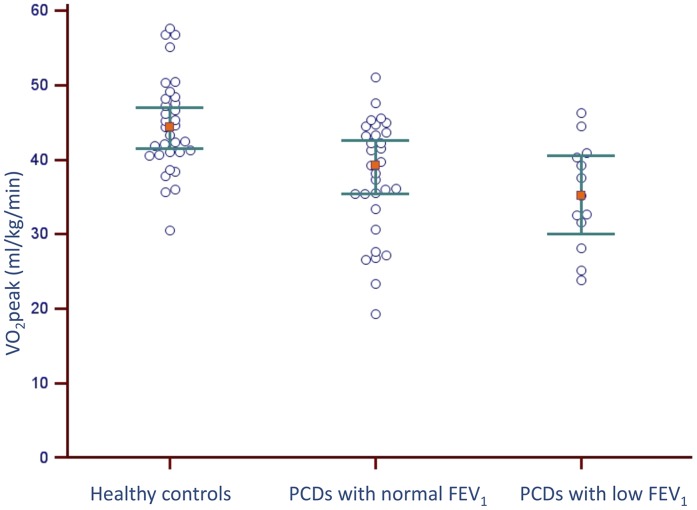
VO_2peak_ in PCD patients with normal FEV_1_ and reduced FEV_1_ (<−1.96 z-score) compared to healthy subjects. Dots are single participants. Red squares are median values with error bars of 95% CI.

The question at hand was whether reduced aerobic fitness was related to impaired lung function. Assuming partial correlations of 0.72 for FEV_1_ z-scores and 0.65 for DL_CO_/V_A_ and α = 0.05, the power exceeds 0.99 for both FEV_1_ z-scores and DL_CO_/V_A_ making the model applicable for further analysis. When adjusting for BMI z-scores, multiple regression analysis using stepwise forward selection, showed that VO_2peak_ was significantly associated with FEV_1_ z-scores (ß-coefficient  = 0.41 z-scores, 95% CI: 0.12–0.70 z-scores; *P* = 0.01) and DLCO/VA (ß-coefficient  = 1.53, 95% CI: 0.24–2.82; *P* = 0.02). For the overall model: Adjusted *R2* = 0.32; *F_3,40_*  = 7.01, *P*<0.01. There was no significant difference in VO_2peak_ between PCD patient subgroups with normal and low (z-score <−1.96) FEV_1_ (Figure 3). Moreover, VO_2peak_ was not associated with whole-body plethysmographic measures (data not shown) or any N_2_ MBW indices (scatter plots in Figure S1). VO_2peak_ did not even differ between patients above or below the 3^rd^ quartile of N_2_ LCI z-scores.

### Self-reported physical activity questionnaire

In the responses to the questions on physical limitations, 34% of patients (15/43) reported being moderately to highly limited, 44% (19/43) slightly limited, and 21% (9/43) not limited at all by sinopulmonary symptoms in activities of everyday-life. In addition, 39% (17/44) of the patients reported moderate to severe limitations in performing vigorous activities, while 30% (13/43) reported only slight difficulties, and 30% (13/43) denied having any difficulties at all.

VO_2peak_ was significantly lower in patients reporting severe limitations in performing vigorous activities compared to in patients without any limitations (*P* = 0.001). VO_2peak_ was also lower in patients who reported that they were highly limited by sino-pulmonary symptoms in everyday-life compared to patients who were not being limited at all (*P* = 0.04).

Responses to questions on weekly physical activity revealed that 30% (13/43) of patients reported performing less than three hours of physical training, while only 16% (7/44) spent more than seven hours every week. VO_2peak_ was significantly correlated with both limitations in everyday activities (*P*<0.01) and vigorous activities (*P*<0.0001). VO_2peak_ was not correlated with self-reported weekly physical activity (*P* = 0.23). None of the healthy controls reported any limitations in physical abilities. Eight of the healthy control subjects reported being physical active les than 5 hours a week, which was not associated with VO_2peak_.

## Discussion

To our knowledge, this is the most comprehensive study to assess VO_2peak_ as well as other common and more sophisticated lung function measures in a large well-characterized group of patients with PCD. We believe that measurement of VO_2peak_ is of utmost relevance for the overall assessment of physical capacity and well-being. As observed in CF, the chronic nature of PCD and the pulmonary impairment has an indisputable effect on the desire and ability to perform physical activity, depending on disease stage. However, little is known about this subject, which prompted us to investigate the potential extent and degree of impairment in aerobic fitness in this rare disease entity.

Children and young adults with PCD, ranging in age from 6–30 years, exhibited significantly lower aerobic fitness than a matched control group. Surprisingly, as many as one third of the PCD patients exhibited significantly and markedly reduced VO_2peak_ compared to the national reference material. This reduction was associated with lower FEV_1_ z-scores and DL_CO_/V_A_, but not with gender, age, or any other lung function parameters, including LCI by N_2_ MBW. VO_2peak_ was also clearly associated with self-reported level of limitations due to difficulties performing vigorous physical activity.

Our finding of reduced aerobic fitness in PCD is consistent with a recently published smaller study by Valerio et al. [12]. They assessed VO_2peak_ in 10 children with PCD and a matched group of eight healthy subjects, and found that VO_2peak_ was significantly reduced in the total group compared to healthy controls; however this reduction was specifically only found in those patients with a reduced FEV_1_ of below 85% of the predicted value. It was not possible to deduce the proportion of patients burdened by abnormal VO_2peak_ z-score. In contrast, Wells et al. [13] performed a study specifically investigating muscle function and metabolism, they reported VO_2 peak_ in 10 patients with PCD, 20 matching healthy controls, and 20 CF patients, but did not find reduced VO_2peak_ in patients with PCD compared to in healthy controls or in CF patients.

There may be several explanations for these contradictory findings. Difference in age is one possibility; both previous studies had a lower mean age ±SD (13.2±2.8 and 13.8±2.3 years, respectively) compared to that in our population (16.9±8.8 years). Differences in gender distribution could also contribute, as there was a higher prevalence of females in our study. However, we did not find any correlation between VO_2peak_ (z-score) and gender or age. Finally, our PCD patients had a median FEV_1_ of 84.9%, which was comparable to those of PCD patients in the study by Valerio et al. [12] (87.5%±9.6%), but somewhat lower compared to those of the patients in the study by Wells et al. [13] (95.4%±9.5%). Studies in CF patients have demonstrated that reduced FEV_1_ is correlated with a low VO_2peak_ [36], although this relationship is non-linear, as CF patients with mild or moderate reduced pulmonary function had normal aerobic fitness compared to CF patients with severely reduced pulmonary function [37].

The mean VO_2peak_ (22.0 ml/kg/min) reported by Valerio et al. [12] is remarkably lower than those in our study (37.4 ml/kg/min) and reported by Wells et al. [13] (41.1 ml/kg/min). There is no clear explanation for this difference. Comparison of results between studies can be difficult due to the clinical heterogeneity of the different patient cohorts, e.g. degree of bronchiectasis and chronic atelectasis. PCD represents a broad spectrum of disease severity, possibly due to multiple mutations [6]; however we did not have sufficient genetic data available to estimate the heterogeneity of our findings. Methodological differences (e.g. choice of exercise protocol, standard operating procedures, and skills of investigators) [38], [39] as well as crude genetic variations in aerobic performances [40] may explain the differences between and within PCD cohorts.

PCD is characterized by obstructive pulmonary impairment as reflected in spirometric and plethysmographic measurements (Table 2). Hence, it was of particular interest to analyze whether this was reflected by ventilatory measures such as V_E_/VCO_2_. As expected, V_E_/VCO_2_ was higher in the PCD group than in healthy controls, and in line with the finding of Valerio et al. [12]. However, remarkable few patients with abnormal VO_2peak_ demonstrated ventilatory limitation as defined by V_E_/VCO_2_ and VR.

Although it has been claimed that VO_2peak_ assessment is the most valid measure of metabolic demand during exercise testing [41], W_max_/kg may also provide additional useful information [42]. We found a significantly reduced W_max_/kg in PCD patients compared to in healthy controls. Abnormal spirometric parameters have consistently been reported in PCD patients [10], [43], [44], [45]. The present data concerning plethysmographic parameters were quite similar to those reported by Pifferi et al. [45], showing significantly increased airway resistance, FRC, RV and RV/TLC. Interestingly, it has been claimed that these measures show better prediction of abnormalities on imaging by chest high-resolution computed tomography than spirometry [45].

The gas-exchanging capacity (DL_CO_ and DL_CO_/V_A_) is a key measurement in interstitial lung diseases, but has been only scarcely reported in PCD patients [46] and, as expected, only a few patients in our study demonstrated abnormal values (<−1.96 SD). Since loss of lung volume due to chronic atelectasis is a common feature in PCD patients, DL_CO_/V_A_ may be a more appropriate parameter than DL_CO_ [45]. DL_CO_/V_A_ appeared to correlate with VO_2peak_, which theoretically seems reasonable, as this functional parameter of the blood-gas interface is an essential factor for sufficient oxygen supply during exercise. However, we do not believe that genuine impairment of pulmonary diffusion plays an important role as a limiting factor for VO_2peak_ in this patient group, since most patients had normal DL_CO_/V_A_ values and normal TLC measured by plethysmography. Measurement of DL_CO_ and estimation of V_A_ during single-breath CO measurement in patients with obstructive disease is problematic, as the uneven ventilation distribution in the short breath-holding time can lead to an incomplete mixing between the inspired gas and the residual gas volume [47]. Additionally, correlation between DL_CO_/V_A_ and VO_2peak_ may depend on a physiological phenomenon, since DL_CO_ (at rest) has been correlated to cardiac output (at rest), which is an important parameter inducible by regular training [48], [49].

Notably, more than 30% of the PCD patients reported limitations in both everyday life and vigorous activities with clear association with the VO_2peak_ level. This finding is comparable to studies in CF patients, that have reported similar association between VO_2peak_ and subjective judgment of physical disability [50]. This association indicates good self-awareness of physical ability and limitations among the patients. In contrast, Valerio et al. [12], surprisingly found that hours spent in vigorous physical activity was not associated with VO_2peak_.

Here we again reported indices of MBW in PCD patients, this time using N_2_ as tracer gas instead of SF_6_ [11]. We consistently observed severe ventilation distribution inhomogeneity, but we found no correlation with VO_2peak_. Interestingly, these parameters did not relate to aerobic fitness even when excessively increased. Further analyses are needed to understand the clinical impact, to compare these observations, and to determine the likely explanation and importance of these findings.

The strengths of the present study include the relatively large and well-characterized PCD cohort and the comprehensive panel of pulmonary function measures reflecting almost all pulmonary functional aspects, – including ventilation distribution inhomogeneity, which has not been previously correlated with VO_2peak_.

To obtain reliable and consistent results, all exercise tests were supervised and conducted by an experienced test leader. Moreover, VO_2peak_ reference values were derived from comparable VO_2peak_ assessments of healthy Danish children and young adults. We proved the robustness of this reference material and the reliability of our test setup by testing a group of healthy subjects with an age range mirroring this material in our laboratory, and we found acceptable agreement between VO_2peak_ levels. Although using a set of pooled data from different age groups and periods as reference may be regarded as a study weakness, it has been documented that VO_2peak_ in healthy subjects is highly repeatable and consistent, both between months [39] and when judged from secular trends provided the tests are performed by highly skilled test leaders using a fixed standard operating procedure for the maximal test [51].

The study was slightly limited by the cross-sectional design and by the use of a non-validated questionnaire on physical limitations and weekly physical or sport activity, which was composed and inspired by questions from previously validated questionnaires. A similar approach was previously used by Pifferi et al. [8], as no validated PCD-specific quality of life questionnaire yet exists. Moreover, activity information derived from self-reported questionnaires is often potentially prone to response bias (e.g. imprecise recall, and influence of social desirability) [52]. However, the questionnaire was completed during the visit but before the exercise test, and in co-operation with the investigator. Corrections and additional information could be provided if necessary to obtain reliable answers. Improved comparison between international studies will require a common validated questionnaire. The BESTCILIA network has been recently developed to provide better diagnostic and treatment tools for PCD, as well as validated PCD-specific quality of life questionnaire, which may satisfy this need [53].

Although we found an association between aerobic fitness and FEV_1_ and DL_CO_/V_A_, it is difficult to estimate the degree of direct influence from these measures. Other important limiting factors that were not measured in the present study include maximal cardiac output, blood oxygen carrying capacity, and peripheral limitations, such as mitochondrial enzyme level and capillary density in muscles [48]. It has not been currently known whether other factors like severity of sinusitis may have any influence, and further studies are needed to explore potential complex association between chronic sinopulmonary disease, cardiopulmonary physiology and VO_2peak_ in PCD.

Despite its limitations, we think that this study provides important new information on the rare pulmonary disease entity PCD. The reduced VO_2peak_ observed in the PCD population might have been caused by the burden of chronic respiratory disease and impairment of pulmonary function, which may lead to a sedentary lifestyle with generally low fitness level, and a theoretically reduced and insufficient capacity to increase cardiac output during vigorous physical activity. This hypothesis is supported by studies in healthy populations that show a sedentary lifestyle to be related to a significantly lower VO_2peak_ [54], [55] and our present findings suggest, that low aerobic fitness may be due to sedentary life style, reflecting the chronic disease. In fact, PCD pulmonary disease might be an additional risk factor of increased morbidity and mortality in these patients since low aerobic fitness is related to increased cardiovascular disease even in otherwise healthy randomly selected children [56]. However, the use of VO_2peak_ as an outcome measure in PCD requires prospective longitudinal studies since longitudinal decline, and not a single VO_2peak_ measurement, might be a better predictor of prognosis, as demonstrated in CF patients [3].

## Conclusion

The present study shows that more than one-third of Danish children and young adults with PCD had significantly abnormal VO_2peak_, which was associated with FEV_1_ z-scores and DL_CO_/V_A_, but not with age or gender. The reduced VO_2peak_ may be related to a sedentary lifestyle caused by the chronic pulmonary disease. We believe that regular physical exercise is of great importance to these patients and should be formally implemented in the clinical management of patients with PCD, with the aim of improving mucociliary clearance and prevent lifestyle-related diseases. Further studies are needed to evaluate VO_2peak_ as a prognostic marker of pulmonary morbidity in PCD.

## Supporting Information

Figure S1 **VO_2peak_ z-scores plotted against indices of N_2_ MBW measurements in patients with PCD.** **A**) VO_2peak_ z-scores vs. N_2_ LCI. **B**) VO_2peak_ z-scores vs. N_2_ S_cond_. **C**) VO_2peak_ z-scores vs. N_2_ S_acin_. VO_2peak_: peak oxygen uptake, LCI: lung clearance index, S_cond_ and S_acin_: normalized phase III slope indices. The dashed red horizontal lines denote the lower limit of normality of VO_2peak_ (mean −1.96 SD). The dashed vertical blue lines denote the upper limits of normal for N_2_ LCI, S_cond_, and S_acin_.
(TIF)

Text S1 **Method for VO_2peak_ measurement.**
(DOC)

Text S2 **Self-reported physical activity questionnaire.** The included questions and the scoring system.
(DOCX)

## References

[1] (1997) Clinical exercise testing with reference to lung diseases: indications, standardization and interpretation strategies. ERS Task Force on Standardization of Clinical Exercise Testing. European Respiratory Society. Eur Respir J 10: 2662–2689. 9426113 10.1183/09031936.97.10112662

[2] Blair SN, Kohl HW III, Paffenbarger RS Jr, Clark DG, Cooper KH, et al (1989) Physical fitness and all-cause mortality. A prospective study of healthy men and women. JAMA 262: 2395–2401. 2795824 10.1001/jama.262.17.2395

[3] Nixon PA, Orenstein DM, Kelsey SF, Doershuk CF (1992) The prognostic value of exercise testing in patients with cystic fibrosis. N Engl J Med 327: 1785–1788. 1435933 10.1056/NEJM199212173272504

[4] Urquhart DS (2011) Exercise testing in cystic fibrosis: why (and how)? J R Soc Med 104 Suppl 1S6–14. 21719895 10.1258/jrsm.2011.s11102 PMC3128161

[5] Barbato A, Frischer T, Kuehni CE, Snijders D, Azevedo I, et al (2009) Primary ciliary dyskinesia: a consensus statement on diagnostic and treatment approaches in children. Eur Respir J 34: 1264–1276. 19948909 10.1183/09031936.00176608

[6] Bush A, Chodhari R, Collins N, Copeland F, Hall P, et al (2007) Primary ciliary dyskinesia: current state of the art. Arch Dis Child 92: 1136–1140. 17634184 10.1136/adc.2006.096958 PMC2066071

[7] McManus IC, Mitchison HM, Chung EM, Stubbings GF, Martin N (2003) Primary ciliary dyskinesia (Siewert's/Kartagener's syndrome): respiratory symptoms and psycho-social impact. BMC Pulm Med 3: 4–16. 14641928 10.1186/1471-2466-3-4 PMC317322

[8] Pifferi M, Bush A, Di CM, Pradal U, Ragazzo V, et al (2010) Health-related quality of life and unmet needs in patients with primary ciliary dyskinesia. Eur Respir J 35: 787–794. 19797134 10.1183/09031936.00051509

[9] Brown DE, Pittman JE, Leigh MW, Fordham L, Davis SD (2008) Early lung disease in young children with primary ciliary dyskinesia. Pediatr Pulmonol 43: 514–516. 18383332 10.1002/ppul.20792

[10] Marthin JK, Petersen N, Skovgaard LT, Nielsen KG (2010) Lung function in patients with primary ciliary dyskinesia: a cross-sectional and 3-decade longitudinal study. Am J Respir Crit Care Med 181: 1262–1268. 20167855 10.1164/rccm.200811-1731OC

[11] Green K, Buchvald FF, Marthin JK, Hanel B, Gustafsson PM, et al (2012) Ventilation inhomogeneity in children with primary ciliary dyskinesia. Thorax 67: 49–53. 21953064 10.1136/thoraxjnl-2011-200726

[12] Valerio G, Giallauria F, Montella S, Vaino N, Vigorito C, et al (2012) Cardiopulmonary assessment in primary ciliary dyskinesia. Eur J Clin Invest 42: 617–622. 22121832 10.1111/j.1365-2362.2011.02626.x

[13] Wells GD, Wilkes DL, Schneiderman JE, Rayner T, Elmi M, et al (2011) Skeletal muscle metabolism in cystic fibrosis and primary ciliary dyskinesia. Pediatr Res 69: 40–45. 20938370 10.1203/PDR.0b013e3181fff35f

[14] Marthin JK, Nielsen KG (2011) Choice of nasal nitric oxide technique as first-line test for primary ciliary dyskinesia. Eur Respir J 37: 559–565. 20525709 10.1183/09031936.00032610

[15] Godfrey S (1970) Physiological response to exercise in children with lung or heart disease. Arch Dis Child 45: 534–538. 5506939 10.1136/adc.45.242.534 PMC1647645

[16] Milani RV, Lavie CJ, Mehra MR, Ventura HO (2006) Understanding the basics of cardiopulmonary exercise testing. Mayo Clin Proc 81: 1603–1611. 17165639 10.4065/81.12.1603

[17] Mahon AD, Marjerrison AD, Lee JD, Woodruff ME, Hanna LE (2010) Evaluating the prediction of maximal heart rate in children and adolescents. Res Q Exerc Sport 81: 466–471. 21268470 10.1080/02701367.2010.10599707

[18] ATS/ACCP Statement on cardiopulmonary exercise testing. Am J Respir Crit Care Med 167: 211–277. 12524257 10.1164/rccm.167.2.211

[19] Andersen LB, Haraldsdottir J (1995) Coronary heart disease risk factors, physical activity, and fitness in young Danes. Med Sci Sports Exerc 27: 158–163. 7723636

[20] Pedersen BK, Andersen LB (2011) Danish Health and Medicines Authority: Physical activity in children and adolescence. Available: http://www.sst.dk/publ/Publ2012/BOFO/FysiskAktivitet/FysiskAktivitet Haandbog.pdf. Accessed 8 February 2013.

[21] Miller MR, Hankinson J, Brusasco V, Burgos F, Casaburi R, et al (2005) Standardisation of spirometry. Eur Respir J 26: 319–338. 16055882 10.1183/09031936.05.00034805

[22] MacIntyre N, Crapo RO, Viegi G, Johnson DC, van der Grinten CP, et al (2005) Standardisation of the single-breath determination of carbon monoxide uptake in the lung. Eur Respir J 26: 720–735. 16204605 10.1183/09031936.05.00034905

[23] Stocks J, Quanjer PH (1995) Reference values for residual volume, functional residual capacity and total lung capacity. ATS Workshop on Lung Volume Measurements. Official Statement of The European Respiratory Society. Eur Respir J 8: 492–506. 7789503 10.1183/09031936.95.08030492

[24] Stanojevic S, Wade A, Stocks J, Hankinson J, Coates AL, et al (2008) Reference ranges for spirometry across all ages: a new approach. Am J Respir Crit Care Med 177: 253–260. 18006882 10.1164/rccm.200708-1248OC PMC2643211

[25] Koopman M, Zanen P, Kruitwagen CL, van der Ent CK, Arets HG (2011) Reference values for paediatric pulmonary function testing: The Utrecht dataset. Respir Med 105: 15–23. 20889322 10.1016/j.rmed.2010.07.020

[26] Zapletal A, Paul T, Samanek M (1977) Significance of contemporary methods of lung function testing for the detection of airway obstruction in children and adolescents (author's transl). Z Erkr Atmungsorgane 149: 343–371. 613549

[27] Kirkby J, Stanojevic S, Welsh L, Lum S, Badier M, et al (2010) Reference equations for specific airway resistance in children: the Asthma UK initiative. Eur Respir J 36: 622–629. 20150205 10.1183/09031936.00135909

[28] Cotes JE, Chinn DJ, Quanjer PH, Roca J, Yernault JC (1993) Standardization of the measurement of transfer factor (diffusing capacity). Report Working Party Standardization of Lung Function Tests, European Community for Steel and Coal. Official Statement of the European Respiratory Society. Eur Respir J Suppl 16: 41–52. 8499053

[29] Quanjer PH, Tammeling GJ, Cotes JE, Pedersen OF, Peslin R, et al.. (1993) Lung volumes and forced ventilatory flows. Report Working Party Standardization of Lung Function Tests, European Community for Steel and Coal. Official Statement of the European Respiratory Society. Eur Respir J Suppl 16: 5–40. 8499054

[30] Robinson PD, Goldman MD, Gustafsson PM (2009) Inert gas washout: theoretical background and clinical utility in respiratory disease. Respiration 78: 339–355. 19521061 10.1159/000225373

[31] Houltz B, Green K, Lindblad A, Singer F, Robinson P, et al.. (2012) Tidal N2 washout ventilation inhomogeneity in a reference population aged 7–70 years (Abstract B3797). Eur Respir J Suppl .

[32] Jones PW, Quirk FH, Baveystock CM (1991) The St George's Respiratory Questionnaire. Respir Med 85 Suppl B: 25–31. 10.1016/s0954-6111(06)80166-6 1759018

[33] Henry B, Aussage P, Grosskopf C, Goehrs JM (2003) Development of the Cystic Fibrosis Questionnaire (CFQ) for assessing quality of life in pediatric and adult patients. Qual Life Res 12: 63–76. 12625519 10.1023/a:1022037320039

[34] Hopkins C, Gillett S, Slack R, Lund VJ, Browne JP (2009) Psychometric validity of the 22-item Sinonasal Outcome Test. Clin Otolaryngol 34: 447–454. 19793277 10.1111/j.1749-4486.2009.01995.x

[35] Ware JE Jr, Sherbourne CD (1992) The MOS 36-item short-form health survey (SF-36). I. Conceptual framework and item selection. Med Care 30: 473–483. 1593914

[36] Pianosi P, Leblanc J, Almudevar A (2005) Relationship between FEV1 and peak oxygen uptake in children with cystic fibrosis. Pediatr Pulmonol 40: 324–329. 16082708 10.1002/ppul.20277

[37] Cropp GJ, Pullano TP, Cerny FJ, Nathanson IT (1982) Exercise tolerance and cardiorespiratory adjustments at peak work capacity in cystic fibrosis. Am Rev Respir Dis 126: 211–216. 7103245 10.1164/arrd.1982.126.2.211

[38] Karila C, de BJ, Waernessyckle S, Benoist MR, Scheinmann P (2001) Cardiopulmonary exercise testing in children: an individualized protocol for workload increase. Chest 120: 81–87. 11451820 10.1378/chest.120.1.81

[39] Andersen LB, Henckel P, Saltin B (1987) Maximal oxygen uptake in Danish adolescents 16–19 years of age. Eur J Appl Physiol Occup Physiol 56: 74–82. 3104033 10.1007/BF00696380

[40] Bouchard C, Dionne FT, Simoneau JA, Boulay MR (1992) Genetics of aerobic and anaerobic performances. Exerc Sport Sci Rev 20: 27–58. 1623888

[41] Wasserman K, Hansen JE, Sue DY, and Whipp BJ (1987) Principles of Exercise Testing and Interpretation. Philadelphia, Lea and febiger.

[42] Gulmans VA, de MK, Binkhorst RA, Helders PJ, Saris WH (1997) Reference values for maximum work capacity in relation to body composition in healthy Dutch children. Eur Respir J 10: 94–97. 9032499

[43] Ellerman A, Bisgaard H (1997) Longitudinal study of lung function in a cohort of primary ciliary dyskinesia. Eur Respir J 10: 2376–2379. 9387968 10.1183/09031936.97.10102376

[44] Noone PG, Leigh MW, Sannuti A, Minnix SL, Carson JL, et al (2004) Primary ciliary dyskinesia: diagnostic and phenotypic features. Am J Respir Crit Care Med 169: 459–467. 14656747 10.1164/rccm.200303-365OC

[45] Pifferi M, Bush A, Pioggia G, Caramella D, Tartarisco G, et al (2012) Evaluation of pulmonary disease using static lung volumes in primary ciliary dyskinesia. Thorax 67: 993–999. 22771515 10.1136/thoraxjnl-2011-200137

[46] Hellinckx J, Demedts M, De BK (1998) Primary ciliary dyskinesia: evolution of pulmonary function. Eur J Pediatr 157: 422–426. 9625342 10.1007/s004310050843

[47] Fitting JW (2004) Transfer factor for carbon monoxide: a glance behind the scene. Swiss Med Wkly 134: 413–418. 15389351 10.4414/smw.2004.10660

[48] Bassett DR Jr, Howley ET (2000) Limiting factors for maximum oxygen uptake and determinants of endurance performance. Med Sci Sports Exerc 32: 70–84. 10647532 10.1097/00005768-200001000-00012

[49] Guazzi M, Reina G, Tumminello G, Guazzi MD (2004) Improvement of alveolar-capillary membrane diffusing capacity with exercise training in chronic heart failure. J Appl Physiol 97: 1866–1873. 15220300 10.1152/japplphysiol.00365.2004

[50] de JW, Kaptein AA, van der Schans CP, Mannes GP, van Aalderen WM, et al (1997) Quality of life in patients with cystic fibrosis. Pediatr Pulmonol 23: 95–100. 9065946 10.1002/(sici)1099-0496(199702)23:2<95::aid-ppul4>3.0.co;2-n

[51] Andersen LB, Froberg K, Kristensen PL, Moller NC, Resaland GK, et al (2010) Secular trends in physical fitness in Danish adolescents. Scand J Med Sci Sports 20: 757–763. 19804576 10.1111/j.1600-0838.2009.00936.x

[52] Shephard RJ (2003) Limits to the measurement of habitual physical activity by questionnaires. Br J Sports Med 37: 197–206. 12782543 10.1136/bjsm.37.3.197 PMC1724653

[53] Bestcilia (2013) Better Experimental Screening and Treatment for Primary Ciliary Dyskinesia. Available at: http://cordis.europa.eu

[54] Rowland TW (1994) Effect of prolonged inactivity on aerobic fitness of children. J Sports Med Phys Fitness 34: 147–155. 7967584

[55] Resaland GK, Andersen LB, Mamen A, Anderssen SA (2011) Effects of a 2-year school-based daily physical activity intervention on cardiorespiratory fitness: the Sogndal school-intervention study. Scand J Med Sci Sports 21: 302–309. 19895384 10.1111/j.1600-0838.2009.01028.x

[56] Anderssen SA, Cooper AR, Riddoch C, Sardinha LB, Harro M, et al (2007) Low cardiorespiratory fitness is a strong predictor for clustering of cardiovascular disease risk factors in children independent of country, age and sex. Eur J Cardiovasc Prev Rehabil 14: 526–531. 17667643 10.1097/HJR.0b013e328011efc1

[57] Olbrich H, Schmidts M, Werner C, Onoufriadis A, Loges NT, et al (2012) Recessive HYDIN Mutations Cause Primary Ciliary Dyskinesia without Randomization of Left-Right Body Asymmetry. Am J Hum Genet 91: 672–684. 23022101 10.1016/j.ajhg.2012.08.016 PMC3484652

