# Supplementary figures and images for: Aerobic Fitness in Children and Young Adults with Primary Ciliary Dyskinesia
Source: PLoS One. 2013 Aug 19;8(8):e71409. doi: 10.1371/journal.pone.0071409 (PMC3747141; doi:10.1371/journal.pone.0071409)

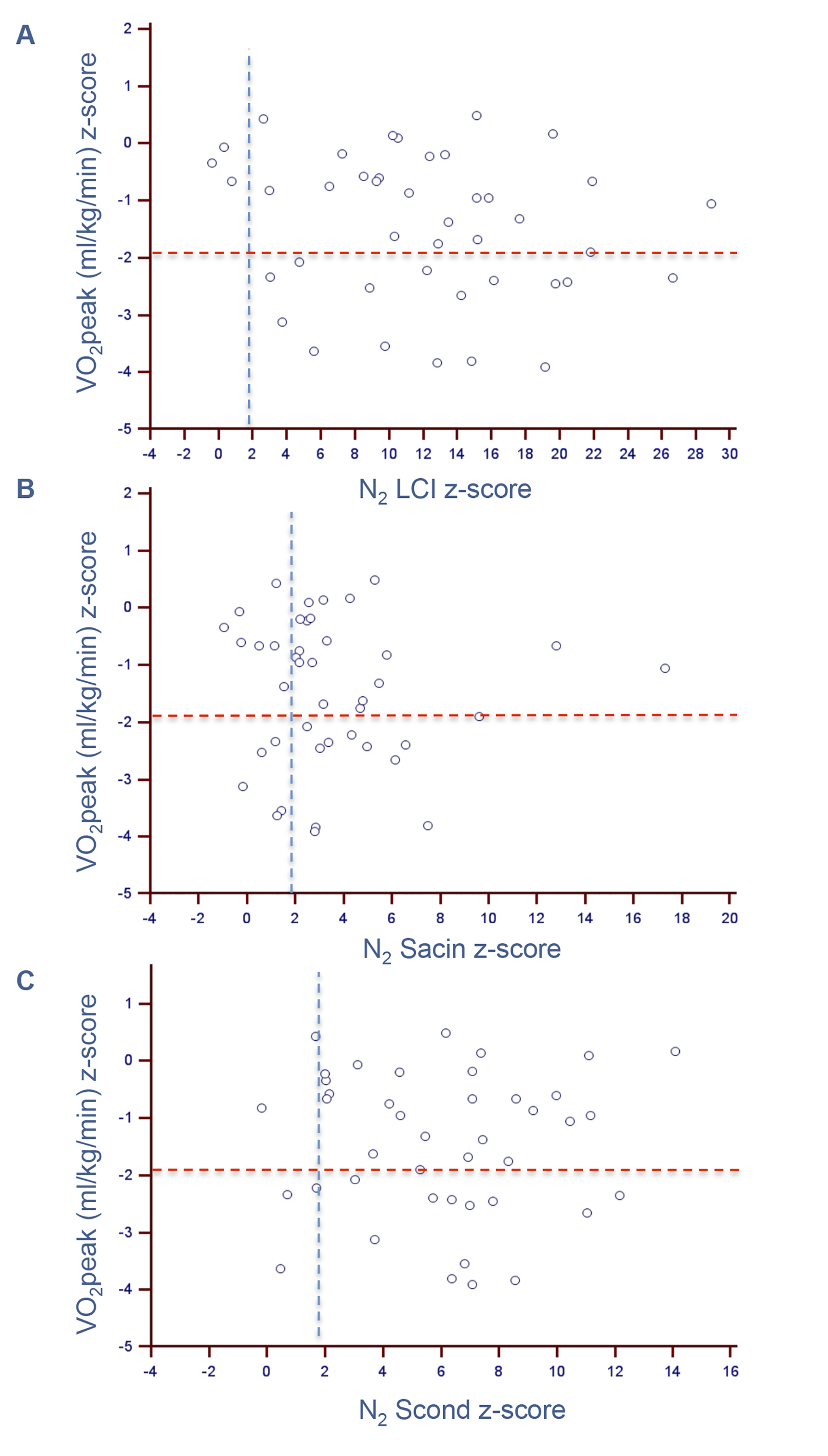

Supplement: Figure S1 — VO2peak z-scores plotted against indices of N2 MBW measurements in patients with PCD. A) VO2peak z-scores vs. N2 LCI. B) VO2peak z-scores vs. N2 Scond. C) VO2peak z-scores vs. N2 Sacin. VO2peak: peak oxygen uptake, LCI: lung clearance index, Scond and Sacin: normalized phase III slope indices. The dashed red horizontal lines denote the lower limit of normality of VO2peak (mean −1.96 SD). The dashed vertical blue lines denote the upper limits of normal for N2 LCI, Scond, and Sacin. (TIF) [file pone.0071409.s001.tif]
