# Supplementary material for: Aerobic Fitness in Children and Young Adults with Primary Ciliary Dyskinesia
Source: PLoS One. 2013 Aug 19;8(8):e71409. doi: 10.1371/journal.pone.0071409 (PMC3747141; doi:10.1371/journal.pone.0071409)
Supplement: Text S1 — Method for VO2peak measurement. (DOC) [file pone.0071409.s002.doc]

Before each test gas and volume calibration was performed using calibration syringes and precision oxygen and carbon dioxide gas mixtures. Patients were asked to refrain from eating two hours prior to exercise test and to wear suitable clothes and shoes. Each patient performed a warm up period followed by a progressive exercise test. To ensure optimal size of cycle ergometer according to age, two different electrically braked cycle ergometers were used (Tecnogym Bike Med D827, Gambettola, Italy and Lode Corrival Pediatric, Intramedic, Groningen, The Netherlands). Patients were strongly encouraged to pedal until exhaustion and to keep a pedal cadence of at least 70 rpm. The patient breathed through a Hans Rudolph mask (Hans Rudolph Inc, KC, USA) connected to a matching breathing valve (2-way NRBV, Hans Rudolph Inc, KC, USA). Four different sizes of masks (pediatric large, adult extra small, adult small and adult large) and two different valves (medium and large) were used to reduce dead space. Expired volume was measured with a breath-by-breath turbine flow meter and a volume transducer, and gas was drawn continuously from the distal end of a mixing chamber. Oxygen saturation (Sp,O_2_) was monitored during exercise testing (BITMOS GmbH, Düsseldorf, Germany) and signs of post-exercise airway obstruction was evaluated by clinical parameters (cough; wheeze, retractions).

Values of minute ventilation (V_E_), tidal volume (VT), respiratory rate (RR), Oxygen uptake (VO_2_), carbon dioxide production (VCO_2_), and respiratory exchange ratio (RER) were obtained every 15 seconds. VO_2_peak (ml/kg/min) was determined as a mean of the highest two consecutive measurements during the last minute of the test. Ventilatory Reserve (VR) was calculated as 1 – (V_E_/MVV)*100, with maximal voluntary ventilation (MVV) defined as FEV1*40.

VO_2_peak reference data was missing for the age of 8, 12 and 20-22. Data for these age groups was constructed by interpolation calculating the mean of the nearest values.
