# Supplementary material for: Aerobic Fitness in Children and Young Adults with Primary Ciliary Dyskinesia
Source: PLoS One. 2013 Aug 19;8(8):e71409. doi: 10.1371/journal.pone.0071409 (PMC3747141; doi:10.1371/journal.pone.0071409)
Supplement: Text S2 — Self-reported physical activity questionnaire. The included questions and the scoring system. (DOCX) [file pone.0071409.s003.docx]

**S2. Self-reported physical activity questionnaire**

Physical limitations

1. To which extend do you find yourself limited in everyday life activities (housekeeping, walking) by your symptoms?

- Not at all (score =1)
- Slightly (score =2)
- Moderately (score = 3)
- Highly (score = 4)

1. To what extend do you have difficulty performing vigorous activities such as running or playing sports?
   - Not at all (score = 1)
   - Slightly (score =2)
   - Moderately (score = 3)
   - Highly (score = 4)

Weekly physical activity (running, cycling and sport)

1. How many hours a week are you physical active?
   - 2 hours or less (score =1)
   - 3-4 hours (score =2)
   - 5-7 hours (score = 3)
   - more than 7 hours (score = 4)
